# Undifferentiated pleomorphic sarcoma of the pancreas: a rare case report and literature review

**DOI:** 10.1186/s12957-022-02525-1

**Published:** 2022-02-27

**Authors:** Ze Liang, Jingzhao Han, Hongfang Tuo, Dongdong Xue, Hanxiang Yu, Yanhui Peng

**Affiliations:** Department of Hepatobiliary, Pancreatic, and Splenic Surgery, Hebei General Hospital, No. 348 Heping West Road, Shijiazhuang, 050000 China

**Keywords:** Pancreatic neoplasms, Undifferentiated pleomorphic sarcoma, Ultrasonography, Treatment, Case report

## Abstract

**Background:**

Primary undifferentiated pleomorphic sarcoma (UPS) of the pancreas is an exceedingly rare malignant tumor, with only 15 cases have been reported in the medical literature. At present, clinicians have poor recognition of the tumor, the epidemiology, diagnosis, and treatment of this disease have yet not been established.

**Case presentation:**

In this report, we depict the clinical and imaging characteristics of a 37-year-old man presenting with a primarily cystic UPS. The patient complained of epigastric pain and distention over 20 days. Abdominal CT and pancreatic magnetic resonance imaging revealed cystic and cystic solid masses in the pancreatic body and tail. An abdominal ultrasound echogram revealed the mass in the body of the pancreas to be cystic with separation echo inside, and the wall was thick, not smooth. Besides, a hypoechoic mass was seen in the tail area of the pancreas with an inhomogeneous echoic pattern, containing small patches of no echo zone in the central. Microscopically, spindle fibroblast-like cells are arranged in a characteristic storiform pattern with pleomorphic and multinucleated cells. Immunohistochemically, tumor cells were positive for CD68 and vimentin. Seven months postoperatively, he was diagnosed with pulmonary lymph node metastasis and died 5 months later. Combined with this case report, we also reviewed the literature regarding UPS of the pancreas.

**Conclusions:**

As we know, this is the first report on ultrasonography findings of pancreatic UPS. Despite there are no distinctive manifestation of UPS, a solid cystic lesion on ultrasonography or a hypodense area in the lesion on T2-weighted imaging, should be considered for differential diagnosis with pancreatic UPS. We believe this article may add some ideas into the diagnosis and therapy of patients with this tumor.

## Background

Undifferentiated pleomorphic sarcoma (UPS) or previously known malignant fibrous histiocytoma (MFH) is considered the most common type of soft tissue sarcoma. It often occurs in the limbs, trunk, and retroperitoneal tissues. However, it has been rarely observed in the digestive organ [1]. Primary pancreatic UPS is an extremely uncommon type of malignant tumor, with only 15 cases have been demonstrated in the medical literature so far. At present, clinicians have poor recognition of the tumor, the epidemiology, diagnosis, and treatment of this disease have yet not been established. Herein, we present a 37-year-old man with UPS of the pancreas with clinical, radiologic, and pathological manifestations. As far as we know, this is the first detailed description of the ultrasonography features of pancreatic UPS.

## Case presentation

**Fig. 1:**
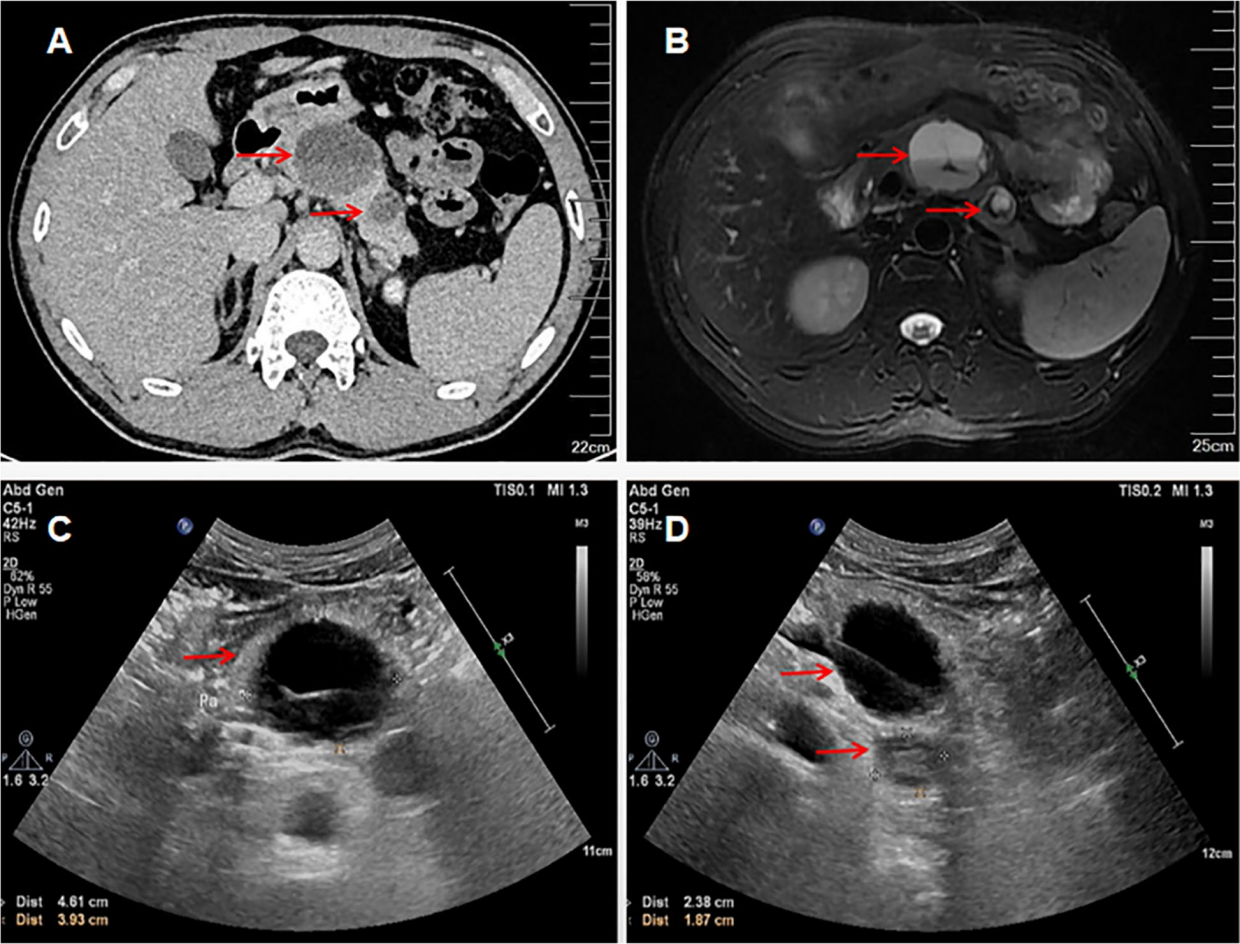
Image of pleomorphic sarcoma of the pancreas. **A** The abdominal CT scan showed two hypointense lesions in the pancreas body and tail. **B** MRI revealed two round cystic abnormal signal involving the body and tail of the pancreas. **C**, **D** Ultrasound imaging exhibited a cystic echo in the body of the pancreas with separation and hypoecho in the tail. (the red arrow indicates the masses)

A 37-year-old man was admitted to our hospital for further evaluation and treatment of pancreatic body and tail masses. He had been suffering from epigastric pain and distention for more than 20 days. The patient had no history of weight loss. The routine hematological laboratory values including tumor markers, hematocrit and liver function tests were within normal limits. He was evaluated by abdominal ultrasonography (US), computed tomography (CT), and pancreatic magnetic resonance imaging (MRI). The abdominal CT scanning revealed two hypodense masses in the body and tail of the pancreas (Fig. 1A). MRI scan of the pancreas confirmed the presence of two rounded cystic mass approximately 4.5 × 4.9 cm and 2.5 × 2.0 cm involving the body and tail of the pancreas respectively, the cystic wall were not smooth. The lesion located in the body appeared as inhomogeneous hyperintensity on T2-weighted images with low intensity septations inside (Fig. 1B). The other mass in the tail whose edge appeared as ring-like low intensity with high intensity in the central part on T2 images. No obvious enhancement in enhanced scanning. An abdominal ultrasound echogram showed the mass in the body of the pancreas to be cystic with separation echo inside, and the wall was thick, not smooth. Besides, a hypoechoic mass was seen in the tail area of the pancreas with an inhomogeneous echoic pattern, containing small patches of no echo zone in the central. No obvious blood flow signal was detected (Fig. 1C, D).

**Fig. 2:**
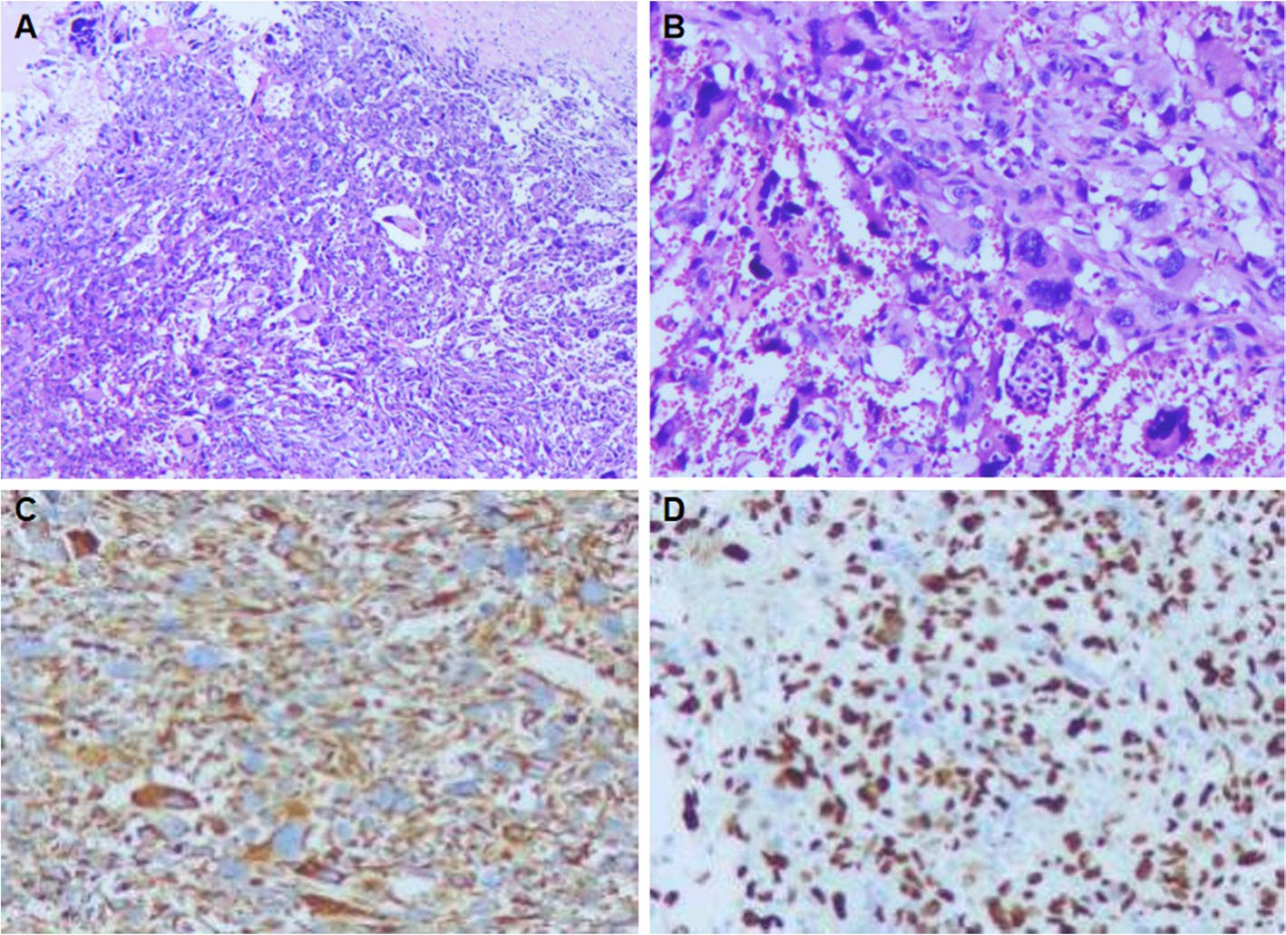
Microscopic section reveals spindle cells arranged in a storiform pattern, with polymorphic neoplastic cells (hematoxylin and eosin, × 40, × 100 original magnification) (**a**, **b**). The cells exhibit the diffuse positive reaction to vimentin (**c**). The cells are positive for p53 (**d**)

After consulting with our hospital’s multiple disciplinary team, the patient was initially diagnosed as benign pancreatic mass and underwent a resection of pancreatic body and tail to relieve the symptom. At laparotomy, two cystic masses were seen in the body and tail of the pancreas measured 5 × 4 cm and 2.5 × 2 cm respectively. The mass was completely resected and the specimen was submitted for pathology evaluation. On pathology, the resected lesion was macroscopically a cystic structure containing sanguineous fluid, the cyst wall was approximately 3 mm wide, and no signs of malignancy were seen. However, microscopic examination demonstrated a malignant neoplasm predominantly consisted of spindle fibroblast-like cells are arranged in a characteristic storiform pattern with pleomorphic and multinucleated cells (Fig. 2). The tumor was 1 cm from the closest pancreas resection margin and the cutting edges of the tumor were negative.

On immunostaining, the tumor was positive for CD68, vimentin and p53 protein, and negative for EMA, CEA, S-100 protein, cytokeratin, Desmin, CD34, CD99, CD117, SMA, MDM2, and CDK4. The tumor Ki-67 expression was about 30%. Based on morphology and immunohistochemical staining, The tumor was diagnosed as UPS. The patient refused to perform further surgery and was discharged 11 days later. Unfortunately, during the follow-up visit at 7 months, a chest CT scan revealed abnormally enlarged lymph nodes. After comparing the preoperative CT scans, those nodes were suspected of being metastatic lesions. The patient died of the metastasis 12 months after surgery.

## Discussion

Primary sarcomas of the pancreas are extremely rare, including leiomyosarcoma, epitheloid sarcomas, granulocytic sarcoma, etc. [2–4]. Pancreatic UPS is one of the rarest primary non-epithelial neoplasms in the pancreas, previously known as MFH [5]. The cells of origin of UPS are believed to be derived from undifferentiated mesenchymocytes, which have the capacity to differentiate into fibroblasts and tissue cells [6]. The pathological characteristics of UPS originating in the pancreas are the same as those in other parts of the body. The majority of cases compose of polymorphy tumors, characterized by cytological and nuclear pleomorphism, mixed with different ratios of spindle cells [7]. They were previously divided into storiform-pleomorphic, inflammatory, giant cell, myxoid, and angiomatoid subtypes [8, 9]. However, in the most recent version of the World Health Organization (WHO) classification, UPS only represents the correct label for the prototypical storiform and pleomorphic variant of MFH [10]. We searched PubMed from the establishment of the database to August 2021, and finally only enrolled 16 cases, including the present case. Therefore, it is important to report the type of tumor morphology for this relatively unknown malignancy.

The clinical profiles of these patients are summarized in Table 1. Among these 16 cases, the ratio of male to female was 12:4. The median age at diagnosis was 57 years. Primary UPS happens to different parts of the pancreas. Nine cases of tumors were located in the body of the pancreas and/or tail of the pancreas, and underwent left pancreatectomy and splenectomy. In 6 cases, it occurred at the pancreatic head and pancreaticoduodenectomy was performed. The preoperative diagnosis of all patients was not clear. Of the 16 cases reported, 11 patients had no further adjuvant therapy. Two patients received adjuvant radiotherapy and chemotherapy and three patients received adjuvant chemotherapy. The longest postoperative follow-up survival time was 48 months.

**Table 1:** Clinical data of the 16 patients with pancreatic UPS

First author	Age	Sex	Histologic type	Location	Treatment	Preoperative diagnosis	Postoperative therapy	Follow-up (months)
Ishiguchi, et al. [11]	44	M	Pleomorphic	Body-tail	Left pancreatectomy, splenectomy	Pancreatic neoplasms	NA	15, NED
Garvey, et al. [12]	77	M	Storiform-pleomorphic	Uncinate lobe	Enucleation	Pancreatic head mass	NA	48, NED
Pascal, et al. [6]	39	M	Storiform-pleomorphic	Head	Pancreaticoduodenectomy	Mesenchymal tumor	NA	0, DOC
Allen, et al. [13]	46	M	Storiform-pleomorphic	Body-tail, local invasion	Pancreatectomy, splenectomy, subtotal gastrectomy	NA	Chemotherapy	5, DOD
Tsujimura, et al. [14]	43	F	Storiform-pleomorphic	Tail	Pancreatectomy, splenectomy	Pancreatic cystadenoma	Chemotherapy	5, NED
Ben Jilani, et al. [15]	72	M	Storiform-pleomorphic	Body-tail	Left pancreatectomy, splenectomy	Pancreatic mass	NA	12, DOD
Balen, et al. [16]	37	M	Pleomorphic	Body-tail	Extended left pancreatectomy	Pancreatic mass	Radiotherapy and chemotherapy	7, DOD
Haba, et al. [17]	70	M	Storiform-pleomorphic	Head	Pancreaticoduodenectomy	Tumor of pancreatic head	Chemotherapy	22, NED
Bastian, et al. [18]	67	M	Storiform-pleomorphic	Body	Left pancreatectomy, splenectomy, transverse colectomy, subtotal gastrectomy	Pancreatic cancer	NA	34, NED
Darvishian, et al. [19]	74	M	Storiform-pleomorphic	Head	Pancreaticoduodenectomy	Pancreatic head cancer	NA	4, NED
Akatsu, et al. [20]	67	M	Storiform-pleomorphic	Body-tail	Left pancreatectomy, splenectomy, transverse colectomy, total gastrectomy	Pancreatic cancer	NA	35, NED
Mizukami, et al. [21]	44	F	Pleomorphic	Body-tail	Total gastrectomy, left pancreatectomy	Pancreatic tumor	NA	20, NED
Yu, et al. [22]	67	M	Storiform-pleomorphic	Head	Pancreaticoduodenectomy	Pancreatic head cyst	NA	11, DOD
Jarry, et al. [7]	45	M	Storiform-pleomorphic	Head	Pancreaticoduodenectomy	Pancreatic cancer	Radiotherapy and chemotherapy	36, NED
Sanei, et al. [1]	72	F	Pleomorphic	Head and neck	Pancreaticoduodenectomy	Pancreatic head cancer	NA	22, NED
Own case	37	F	Pleomorphic	Body-tail	Distal pancreatectomy	Pancreatic mass	NA	12, DOD

Primary pancreatic UPS is more prevalent in men than in women. However, due to the limited number, other risk factors have not been determined. It grows fast, and its clinical features described in literature are varied, but upper abdominal discomfort is the most common presentation. At present, there is no reliable laboratory test contributing to diagnosis, and the preoperative tumor markers in this case are negative. In the previous reports, pancreatic UPS presented a large, nonhomogeneous, hypointense or multinodular lesion with possible intratumoral calcification [23] and massive liquescent necrosis in CT plain scan [18, 20]. Enhanced modalities showed a non-homogeneously enhancing mass with enhancing peripheral pseudocapsular [22]. The case reported by Yu et al. [22] showed a huge multilocular cystic lesion on abdominal CT and MRI, which contained a large amount of liquefaction necrosis, and the cyst wall, fibrous septum, and solid components were enhanced. Miller et al. [24] found that internal low-intensity separation on MRI and nonhomogeneous high-intensity on T2-weighted images were the general characteristics of malignant soft tissue tumors. The preoperative CT and MRI of our patient showed cystic or cystic solid pancreatic masses with blood deposition. A low signal region on T2-weighted images that reflected the fibrous element was also observed in our patient, which was similar to the case reported by Yu et al., but there was no obvious enhancement in each phase. As we know, we depict the first case of primary UPS of the pancreas with ultrasound examination findings. The author believes that for ultrasound diagnosis of cystic solid pancreatic mass, the possibility of malignant lesions should be considered for those with thick and uneven wall thickness and mixed solid echo and cystic echo within the lesion.

In general, it is difficult to diagnose pancreatic UPS without obtaining tumor tissues. Endoscopic ultrasonography-guided needle aspiration has become the most accurate method for the diagnosis of pancreatic malignant tumors [5]. However, in order to prevent tumor spread and needle implantation, a routine preoperative biopsy is not recommended. For patients who are difficult to identify and require radiotherapy or chemotherapy, puncture can be performed for pathological examination. Immunohistochemical examination can rule out other types of sarcoma and ultimately confirm the diagnosis of UPS. The pathological differential diagnosis among the pancreatic neoplasms includes leiomyosarcomas, liposarcomas, neurogenic sarcomas, and malignant lymphomas which have respective tumor markers [20, 25, 26]. UPS is reserved only for those soft tissue sarcomas which do not show any definitive line of differentiation. The present case showed positive staining for vimentin, CD68, and p53, negative for S-100, SMA, desmin, or CEA was observed, which differentiates this neoplasm from other sarcomas. Previous studies have pointed out that UPS exhibits strong responses to vimentin and CD68 as this case did [20, 27]. Mizukami et al. [21] concluded that positivity for p53 in the tumor cells may be helpful in distinguishing the UPS from pseudotumors such as inflammatory pseudotumors.

Surgery is the mainstay of treatment in primary localized retroperitoneal sarcomas [28]. Primary pancreatic UPS appears to parallel the biological behavior of retroperitoneal UPS. Thus, the therapy of pancreatic UPS can follow the treatment principles of the latter. Radical resection is the mainstay method and the choice of surgical method depends on the location of the tumor. Due to the short follow-up period of previous studies, the final prognosis after surgical excision is hard to evaluate. In the case here described, the preoperative laboratory examination and intraoperative gross specimens revealed benign lesions. Considering that the patient was young, pancreatic body and tail resection was performed to preserve pancreatic function. However, postoperative pathological examination and immunohistochemistry confirmed the diagnosis of pancreatic primary UPS. The poor outcome of the current case may be due to incomplete resection as well as to the biological malignant potential. According to Sugita et al the Ki-67 labeling index has a negative correlation with the prognosis of soft tissue tumors [29]. This corresponds to our case presented with a high Ki-67 labeling index score. Through the diagnosis and treatment of this patient, we have learned many lessons: (1) although rare, UPS of pancreas is essential to consider when making a differential diagnosis in a patient with a cystic solid pancreatic lesion. (2) For patients who fail to obtain histological examination before operation, an intraoperative frozen examination should be carried out as much as possible to reduce the missed diagnosis of pancreatic malignant tumors. (3) If the initial surgery is not complete, radical surgery should be performed again in time to achieve an R0 situation.

In addition to surgery, radiotherapy may have a certain effect on pancreatic UPS. Studies have found that when the tumor cannot be completely removed, radiotherapy is an important adjuvant treatment for UPS in other parts [30]. However, due to the limited number, the role of adjuvant radiotherapy on these tumors has not been determined. Chemotherapy has been reported to effectively prolong survival for patients with UPS in the soft tissue. Doxorubicin and ifosfamide are usually recommended as the first-line chemotherapy regimen for soft tissue sarcoma [31], but based on the existing literature, which regimen should be adopted for pancreatic UPS are still being explored. Jarry et al. [7] report a case of resected pancreatic UPS who recurred 11 months later presenting as pulmonary and hepatic metastasis. The patient underwent a multidisciplinary therapy of radiofrequency ablation, chemotherapy, and a right hepatectomy. Then, the patient recovered completely and was disease-free for 3 years after the operation. It seems that such treatment could improve the survival rates of recurring patients.

## Conclusion

In summary, primary UPS is an exceptionally rare but distinct malignant neoplasm of pancreas. Despite there are no distinctive manifestation of UPS, a solid cystic lesion on ultrasonography or a hypodense area in the lesion on T2-weighted imaging, should be considered for differential diagnosis with pancreatic UPS. Radical resection is the most effective treatment at present. Postoperative radiotherapy and chemotherapy may improve the survival of patients, which needs to be verified by more clinical cases. This case emphasizes the clinicopathological, imaging, and immunohistochemical finding of pancreatic UPS. Long-term follow-up researches are required to know the exact biological behavior of these neoplasms.

## Data Availability

All data generated during this study are included in this published article.

## Abbreviations

UPS: Undifferentiated pleomorphic sarcoma
MFH: Malignant fibrous histiocytoma
CT: Computed tomography
US: Ultrasonography
MRI: Pancreatic magnetic resonance imaging
WHO: World Health Organization

## References

[1] Sanei B, Kefayat A, Samadi M, Goli P, Sanei MH, Ghahremani F (2018). Undifferentiated pleomorphic sarcoma of pancreas: a case report and review of the literature for the last updates. Case Rep Med.

[2] Ambe P, Kautz C, Shadouh S, Heggemann S, Köhler L (2011). Primary sarcoma of the pancreas, a rare histopathological entity. A case report with review of literature. World J Surg Oncol.

[3] Messager M, Amielh D, Chevallier C, Mariette C (2012). Isolated granulocytic sarcoma of the pancreas: a tricky diagnostic for primary pancreatic extramedullary acute myeloid leukemia. World J Surg Oncol.

[4] Xu J, Zhang T, Wang T, You L, Zhao Y (2013). Clinical characteristics and prognosis of primary leiomyosarcoma of the pancreas: a systematic review. World J Surg Oncol.

[5] Okasha HH, Naga MI, Esmat S, Naguib M, Hassanein M, Hassani M (2013). Endoscopic ultrasound-guided fine needle aspiration versus percutaneous ultrasound-guided fine needle aspiration in diagnosis of focal pancreatic masses. Endosc Ultrasound.

[6] Pascal RR, Sullivan L, Hauser L, Ferzli G (1989). Primary malignant fibrous histiocytoma of the pancreas. Hum Pathol.

[7] Jarry J, Belleannee G, Laurent C, Coindre JM, Evrard S (2010). Primary malignant fibrous histiocytoma of the pancreas: benefit of the multidisciplinary approach. Eur J Gastroenterol Hepatol.

[8] Scapolan M, Perin T, Wassermann B, Canzonieri V, Colombatti A, Italia F (2008). Expression profiles in malignant fibrous histiocytomas: clues for differentiating ‘spindle cell’ and ‘pleomorphic’ subtypes. Eur J Cancer.

[9] Fletcher CD (2014). The evolving classification of soft tissue tumours - an update based on the new 2013 WHO classification. Histopathology..

[10] Sbaraglia M, Bellan E, Dei Tos AP (2021). The 2020 WHO Classification of Soft Tissue Tumours: news and perspectives. Pathologica..

[11] Ishiguchi T, Shimamoto K, Kaii O, Sakai M, Ishigaki T, Sakuma S (1986). Malignant fibrous histiocytoma of the pancreas. Rinsho Hoshasen.

[12] Garvey JF, Ng A, England JF, Sheldon DM (1989). Malignant fibrous histiocytoma of the pancreas. HPB Surg.

[13] Allen KB, Skandalakis LJ, Brown BC, Gray SW, Skandalakis JE (1990). Malignant fibrous histiocytoma of the pancreas. Am Surg.

[14] Tsujimura T, Kawano K, Taniguchi M, Yoshikawa K, Tsukaguchi I (1992). Malignant fibrous histiocytoma coexistent with mucinous cystadenoma of the pancreas. Cancer..

[15] Ben Jilani S, el Mezni F, Dziri C, Khatteche A, Zermani R, Najeh N (1993). Primary malignant histiocytofibroma of the pancreas. Apropos of a case. Arch Anat Cytol Pathol.

[16] Balén EM, De Villa VH, Cienfuegos JA, Contreras F, Pardo F, González J (1993). Malignant fibrous histiocytoma of the pancreas. Rev Esp Enferm Dig.

[17] Haba R, Kobayashi S, Hirakawa E, Miki H, Okino T, Kurokawa T (1996). Malignant fibrous histiocytoma of the pancreas. Pathol Int.

[18] Bastian D, Ramaswamy A, Barth PJ, Gerdes B, Ernst M, Bartsch D (1999). Malignant fibrous histiocytoma of the pancreas: a case report with genetic analysis. Cancer..

[19] Darvishian F, Sullivan J, Teichberg S, Basham K (2002). Carcinosarcoma of the pancreas: a case report and review of the literature. Arch Pathol Lab Med.

[20] Akatsu T, Tsugita M, Ro S, Kameyama K, Kitajima M (2005). Primary malignant fibrous histiocytoma of the pancreas: a case with K-ras mutation and a review of the literature. Dig Dis Sci.

[21] Mizukami H, Yajima N, Wada R, Matsumoto K, Kojima M, Klöppel G (2006). Pancreatic malignant fibrous histiocytoma, inflammatory myofibroblastic tumor, and inflammatory pseudotumor related to autoimmune pancreatitis: characterization and differential diagnosis. Virchows Arch.

[22] Yu RS, Wang JW, Chen Y, Ding WH, Xu XF, Chen LR (2008). A case of primary malignant fibrous histiocytoma of the pancreas: CT and MRI findings. World J Gastroenterol.

[23] Ko SF, Wan YL, Lee TY, Ng SH, Lin JW, Chen WJ (1998). CT features of calcifications in abdominal malignant fibrous histiocytoma. Clin Imaging.

[24] Miller TT, Hermann G, Abdelwahab IF, Klein MJ, Kenan S, Lewis MM (1994). MRI of malignant fibrous histiocytoma of soft tissue: analysis of 13 cases with pathologic correlation. Skeletal Radiol.

[25] Coindre JM, Mariani O, Chibon F (2003). Most malignant fibrous histiocytomas developed in the retroperitoneum are dedifferentiated liposarcomas: a review of 25 cases initially diagnosed as malignant fibrous histiocytoma. Mod Pathol.

[26] Wardelmann E, Schildhaus HU, Merkelbach-Bruse S, Hartmann W, Reichardt P, Hohenberger P (2010). Soft tissue sarcoma: from molecular diagnosis to selection of treatment. Pathological diagnosis of soft tissue sarcoma amid molecular biology and targeted therapies. Ann Oncol.

[27] Ye MF, Zheng S, Xu JH, Chen LR (2007). Primary hepatic malignant fibrous histiocytoma: a case report and review of the literature. Histol Histopathol.

[28] Novak M, Perhavec A, Kerin Povšič M, Arnuš M, Eržen D (2020). Primary localized retroperitoneal sarcomas: report from Slovenian sarcoma referral center. World J Surg Oncol.

[29] Sugita S, Segawa K, Kikuchi N, Takenami T, Kido T, Emori M (2022). Prognostic usefulness of a modified risk model for solitary fibrous tumor that includes the Ki-67 labeling index. World J Surg Oncol.

[30] Sheplan LJ, Juliano JJ (2010). Use of radiation therapy for patients with soft-tissue and bone sarcomas. Cleve Clin J Med.

[31] Lorigan P, Verweij J, Papai Z, Rodenhuis S, Le Cesne A, Leahy MG (2007). European Organisation for Research and Treatment of Cancer Soft Tissue and Bone Sarcoma Group Study. Phase III trial of two investigational schedules of ifosfamide compared with standard-dose doxorubicin in advanced or metastatic soft tissue sarcoma: a European Organisation for Research and Treatment of Cancer Soft Tissue and Bone Sarcoma Group Study. J Clin Oncol.

